# Active Construction of Profession-Related Events: The Priming Effect among Pre-service Teachers with Different Professional Identity

**DOI:** 10.3389/fpsyg.2018.00233

**Published:** 2018-02-27

**Authors:** Xin-qiang Wang, Jun-cheng Zhu, Lu Liu, Xiang-yu Chen, Jun-yu Huo

**Affiliations:** School of Psychology, Center for Mental Health Education and Research, Jiangxi Key Laboratory of Psychology and Cognition Science, Jiangxi Normal University, Nanchang, China

**Keywords:** pre-service teacher, teacher professional identity, profession-related event, active construction, priming effect, self-schema

## Abstract

Pre-service teachers with different professional identity may actively construct different subjective profession-related events based on the same objective profession-related events. To explore the priming effect among pre-service teachers with different professional identity, this study examined the effect of positive, negative, or neutral priming sentences in an individualized narration of profession-related events through a priming paradigm. Forty-two female volunteers were asked to complete positive, negative, and neutral priming sentences describing profession-related events. The results showed that, relative to those with weak professional identity, participants with strong professional identity generated a higher number of positive items when primed with different stimuli and displayed greater positive priming bias for positive and neutral stimuli. In addition, relative to those with strong professional identity, participants with weak professional identity generated a higher number of neutral and negative items when primed with positive and negative stimuli, respectively, and displayed greater negative priming bias toward negative stimuli. These results indicate that pre-service teachers with strong professional identity were likely to have established positive self-schemas involving profession-related events, which facilitated active, positive construction of such events.

## Introduction

In the field of education, pre-service teachers are the main source of future teachers, and their professional identity is a longstanding topic of concern in all sectors of society (Akkerman and Meijer, 2011; Ma et al., 2013; Wei et al., 2013; Stenberg et al., 2014). Pre-service teachers’ professional identity refers to their positive attitudes toward the teaching profession and their current identity as pre-service teachers (Wang et al., 2017). The formation of this identity involves a process of dynamic change and continuous development (Hong, 2010; Trent, 2011; Izadinia, 2013; Pillen et al., 2013). In other words, the strength of pre-service teachers’ professional identity is influenced by several internal factors, such as motivation and emotions (Timoštšuk and Ugaste, 2012; Mancini et al., 2015), and external factors, such as environment, policies, education, and previous experience (Pinho and Andrade, 2015; Zhao and Zhang, 2015). In addition, many researchers have shown that, while teachers’ professional identity is a continuous process of dynamic development, it exhibits certain relatively stable features, which are developed mainly during pre-service teacher education (Sutherland et al., 2010; Izadinia, 2013).

Research on teachers’ professional identity is not a new theme; however, research examining pre-service teachers’ professional identity is in the preliminary stage (Feng et al., 2010; Timoštšuk and Ugaste, 2010). Most studies have focused on examining pre-service teachers’ professional identity from the perspectives of theoretical discourse, interviews and observation, product analysis, questionnaire preparation, and surveys concerning current status and influential factors. However, very few experimental studies have been conducted to examine this topic (Izadinia, 2013; Ma et al., 2013; Akerson et al., 2014; Fan et al., 2014; Zhao and Zhang, 2015). Surveys have shown that teachers with stronger professional identity were more likely to maintain their enthusiasm toward teaching, exhibited greater job satisfaction, and were less likely to display job burnout or turnover intention, relative to those with weak professional identity (Wei and Song, 2012; Fisherman, 2014; Luo et al., 2014). Moreover, some studies showed that pre-service teachers with strong professional identity were proactive in overcoming poor learning conditions and improving their academic performance (Zhang et al., 2011; Abednia, 2012). In contrast, pre-service teachers with weak professional identity exhibited poorer academic and life satisfaction and greater concern regarding their future, relative to that observed in those with strong professional identity (Wang et al., 2011). The above-mentioned findings indicate that examination of pre-service teachers’ professional identity could not only enrich and extend current research in this regard, but also facilitate the cultivation of pre-service teachers’ professional psychological qualities, thereby promoting their overall development.

The results of multiple surveys showed that pre-service teachers’ overall professional identity was relatively strong (Feng et al., 2010; Wu et al., 2010; Zhao and Zhang, 2015). However, the following questions remain unanswered: (1) Do the types of social information processing adopted when faced with positive, negative, or neutral events in their teaching careers differ between pre-service teachers with strong and weak professional identity? and (2) Why do pre-service teachers with strong professional identity display better learning motivation, career preparation, professional development, and mental health, relative to that observed in pre-service teachers with weak professional identity? Therefore, research including a control group of pre-service teachers with weak professional identity is required, to allow in-depth investigation of the effects of priming involving events encountered in teaching in pre-service teachers with strong professional identity. This could also provide empirical evidence for the cultivation of professional identity in pre-service teachers, by revealing differences in the internal psychological mechanisms underlying social information processing between pre-service teachers with strong and weak professional identity.

Social information processing in individuals with depression and internet addiction has been examined via the priming paradigm used in Tversky’s social cognition experiment (Tversky and Marsh, 2000; Feng et al., 2008; Wang et al., 2008). Tversky’s social-cognitive experimental paradigm aims to test cognitive processes about life events using experimental procedures such as the “Roommates Story” protocol. In these paradigms, participants are asked to write down their own thoughts immediately after reading a priming stimulus. Meanwhile, Wyer and Srull (1989) posited that the priming effect is induced by stimuli that activate behavior-related concepts at the top of one’s lexicon. In contrast to other priming paradigms, such as the Stroop and dot-probe tasks, this paradigm allows for the quantitative manipulation of profession-related and other events (Yu et al., 2015). Therefore, it could be effective in evaluating social information processing in pre-service teachers primed with different types of profession-related events. Moreover, survey studies have demonstrated differences in professional development and mental health between pre-service teachers with strong and weak professional identity. Therefore, the effects of priming involving events encountered during teaching careers could differ between these two groups, which could reveal important internal psychological mechanisms that cause pre-service teachers to shift toward positive or negative development. Based on the above-mentioned findings and assumptions, we established the following research hypothesis: Relative to pre-service teachers with weak professional identity, pre-service teachers with strong professional identity will generate a higher number of positive items pertaining to different types of profession-related events and exhibit positive priming bias. In other words, pre-service teachers with strong professional identity will show higher levels of positively primed processing; generate a higher number of positive items when primed with positive, neutral, and negative stimuli; and exhibit greater positive priming bias toward positive stimuli.

## Materials and Methods

### Participants

The Student Teacher Professional Identity Scale developed by Wang et al. (2010, 2017) was administered to 300 female volunteers randomly selected from junior students studying education at Jiangxi Normal University, to examine the strength of their professional identity. The scale includes 12 items divided between the following four dimensions: professional values (three items; e.g., “I think that student teachers are respected”), professional efficacy (three items; e.g., “I have the ability to master teaching skills”), professional willingness (three items; e.g., “I am willing to communicate with pupils”), and professional volition (three items; e.g., “I will be a teacher for life”). Responses are provided using a five-point scale ranging from 1 (*strongly disagree*) to 5 (*strongly agree*), and reverse-scored questions are recoded before calculating total scores. Higher scores indicate stronger professional identity. Cronbach’s α for the scale was 0.85 in the current study. The participants were assigned anonymous identification codes for use during the entire study. Participants were ranked in descending order according to their total scores, and the top and bottom 27% of the population were selected. Following this procedure, 42 female volunteers were willing to take part in this study. Among them, 21 participants with the highest professional identity scores were assigned to the strong professional identity group (*M*_high_ = 49.14, *SD*_high_ = 3.20), and 21 participants with the lowest professional identity scores were assigned to the weak professional identity group (*M*_low_ = 37.00, *SD*_low_ = 3.55). Participants’ ages ranged between 19 and 24 (*M* = 21.24, *SD* = 1.34) years. There was a significant difference in level of professional identity between the strong and weak professional identity groups, *t*(40) = 11.67, *p* < 0.001, *Cohen’s d* = 3.68.

### Experimental Design

Differences in priming effects between students with strong and weak professional identity were examined via the priming paradigm used in Tversky’s social cognition experiment (Tversky and Marsh, 2000; Feng et al., 2008; Wei, 2008). A 2 (professional identity: strong, weak) × 3 (priming stimulus: positive, negative, neutral) two-way mixed study design was used, with professional identity as the between-subjects variable and priming stimulus as the within-subjects variable. The number of items (i.e., positive, negative, or neutral) generated by participants following priming with the positive, negative, or neutral stimuli was used as the dependent variable. The judgment criteria for the generated items and priming stimuli were identical. Specifically, positive priming stimuli and generated items referred to proactivity and optimism; neutral priming stimuli and generated items referred to objectivity and emotional neutrality; and negative priming stimuli and generated items referred to passivity and pessimism. Overall judgment of the types of stimuli used in priming was conducted independently by six individuals, including two individuals with doctoral degrees in psychology engaged in research in educational psychology, three individuals with master’s degrees in psychology (two of whom were highly experienced and in service at secondary and primary schools), and one undergraduate student in teacher training. The level of consensus between the six raters was 86.6%, which indicated good inter-rater agreement.

### Experimental Materials

The reading material was the “Colleague Story” prepared by Wei (2008). This story describes events (e.g., taking classes, lesson preparation, teaching, and salaries) that occur during the teaching careers of two colleagues (Supplementary Presentation 1). The priming stimuli included 54 incomplete sentences (18 positive, 18 neutral, and 18 negative) describing profession-related events for the two colleagues (e.g., “The students really like Mr. Zhang, Mr. Zhang will ____”; Wei, 2008).

We assessed the interitem consistency of the different priming stimuli to determine types, presentation frequency, and homogeneity. The results indicated that the inter-item consistency values for the positive, neutral, and negative stimuli were 0.80, 0.71, and 0.81, respectively; therefore, the material met the measurement requirements for psychological experimentation.

### Experimental Procedure

The study was approved by the ethics committee of the unit. Participants completed the Student Teacher Professional Identity Scale (Wang et al., 2010, 2017) in class, to identify those with low and high professional identity scores. One week later, 42 female volunteers agreed to come to the lab and completed the experiment. First, all participants were asked to sign an informed consent form and complete a demographic information survey. Second, all participants were assigned to complete the priming task used in Tversky’s social cognition experiment (Tversky and Marsh, 2000; Feng et al., 2008; Wei, 2008). They read the Colleague Story within 5 min and returned the reading material to the researcher. The participants were then asked to count backwards in threes from 500 before proceeding to the priming test phase. During the priming test phase, the researcher presented participants with the 54 incomplete sentences, used as primers for the Colleague Story. The participants were asked to complete all of the sentences within 20 min.

### Data Analysis

The processing capacity values for positive, neutral, and negative priming stimuli were calculated as the sums of all positive, neutral, and negative items generated, respectively, under the priming effects of positive, neutral, and negative stimuli. The results of tests of normality showed no significant skewness (*p* > 0.05), indicating that the data were normally distributed, and the results of the test of homogeneity of variance showed no significant homogeneity (*p* > 0.05). In addition, differences in the numbers of items generated by participants between the three types of priming stimuli were significant *F*(2,123) = 85.54, *p* < 0.001, which showed the validity of the priming stimulus. Therefore, we performed a 2 (professional identity: weak, strong) × 3 (priming stimulus: positive, neutral, negative) repeated measures analysis of variance (ANOVA) to assess participants’ processing capacity for different types of priming stimuli. In addition, we performed a second 2 (professional identity: weak, strong) × 3 (items generated: positive, neutral, negative) repeated measures ANOVA to examine participants’ positive priming bias.

## Results

### Differences in Priming Effects According to Participants’ Professional Identity

**FIGURE 1:**
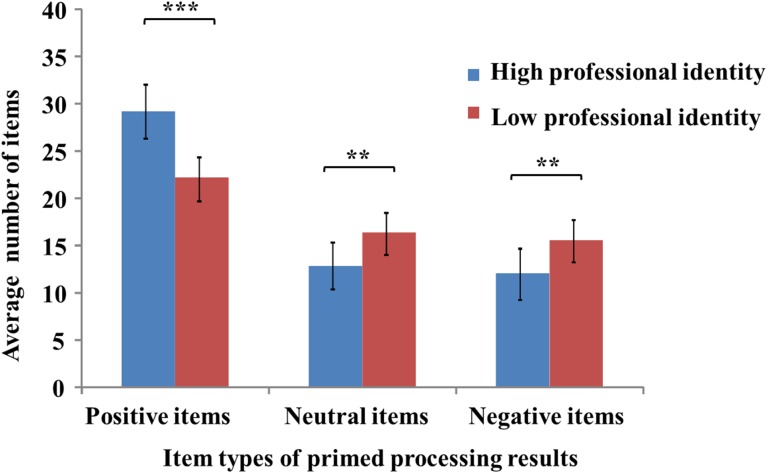
Primed capacity for positive, neutral, and negative stimuli in pre-service teachers with strong and weak professional identity. ^∗∗^*p* < 0.01, ^∗∗∗^*p* < 0.001.

The results of the first ANOVA indicated that the main effects of priming stimulus, *F*(2,40) = 78.44, *p* < 0.001, *partial η^2^* = 0.66, and professional identity, *F*(1,40) = 6.66, *p* < 0.05, *partial η^2^* = 0.26, and the interaction between these two variables, *F*(2,80) = 16.39, *p* < 0.001, *partial η^2^* = 0.29, were significant. Simple effects analysis showed that pre-service teachers with strong professional identity generated higher numbers of positive items for all stimulus types during primed processing, relative to those observed for pre-service teachers with weak professional identity (*p* < 0.001; **Figure 1**). In addition, pre-service teachers with weak professional identity generated higher numbers of negative and neutral items for all types of stimulus during primed processing, relative to those observed for pre-service teachers with strong professional identity (*p* < 0.001). These results indicated that pre-service teachers with strong professional identity showed a high level of positively primed processing, while pre-service teachers with weak professional identity showed high levels of neutrally and negatively primed processing.

### Differences in Processing Capacity for Positive, Neutral, and Negative Priming Stimuli According to Participants’ Professional Identity

Further analysis indicated that processing capacity for positive, neutral, and negative priming stimuli differed between pre-service teachers with weak and strong professional identity (**Table 1**). Following priming with positive stimuli, pre-service teachers with strong professional identity generated a significantly higher number of positive items, *t*(40) = 4.86, *p* < 0.001, *Cohen’s d* = 1.54, and a significantly lower number of neutral items, *t*(40) = -3.97, *p* < 0.001, *Cohen’s d* = -1.26, relative to those observed for pre-service teachers with weak professional identity; however, the numbers of negative items generated did not differ significantly between the two groups (*p* > 0.05). Following priming with neutral stimuli, pre-service teachers with strong professional identity generated a significantly higher number of positive items relative to that observed for those with weak professional identity, *t*(40) = 3.00, *p* < 0.001, *Cohen’s d* = 0.95; however, the numbers of neutral and negative items generated did not differ significantly between the two groups (*p* > 0.05). Following priming with negative stimuli, pre-service teachers with strong professional identity generated a significantly higher number of positive items, *t*(40) = 3.59, *p* < 0.001, *Cohen’s d* = 1.14, and a significantly lower number of negative items, *t*(40) = -2.29, *p* < 0.05, *Cohen’s d* = -0.72, relative to those observed for pre-service teachers with weak professional identity; however, the numbers of neutral items generated did not differ significantly between the two groups (*p* > 0.05).

**Table 1:** Processing capacity for positive, neutral, and negative priming stimuli in pre-service teachers with strong and weak professional identity.

Stimulus type	Generated item	Weak professional identity (*n* = 21)		Strong professional identity (*n* = 21)		*T*
		*M*	*SD*	*M*	*SD*	
Positive stimulus	Positive items	14.86	1.68	16.86	0.85	4.86^∗∗∗^
	Neutral items	2.62	1.83	0.95	0.59	-3.97^∗∗∗^
	Negative items	0.52	1.21	0.19	0.40	-1.20
Neutral stimulus	Positive items	4.33	2.20	6.62	2.71	3.00^∗∗∗^
	Neutral items	9.86	2.18	8.52	2.87	-1.70
	Negative items	3.81	1.54	2.86	1.62	-1.95
Negative stimulus	Positive items	2.95	1.53	5.67	3.10	3.59^∗∗∗^
	Neutral items	3.86	2.13	3.33	2.03	-0.82
	Negative items	11.19	2.48	9.01	3.62	-2.29^∗^

*^∗^p < 0.05, ^∗∗∗^p < 0.001.*

### Difference in Priming Bias According to Participants’ Professional Identity

**FIGURE 2:**
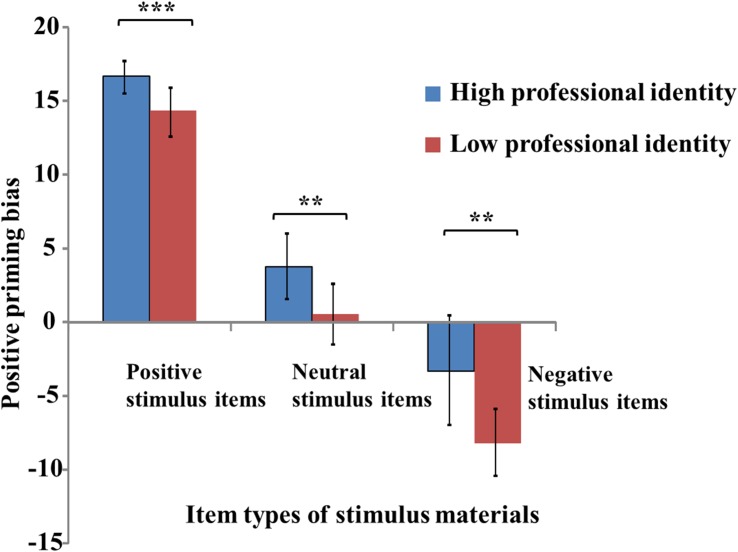
Positive priming bias toward different stimulus types in pre-service teachers with strong and weak professional identity. ^∗∗^*p* < 0.01, ^∗∗∗^*p* < 0.001.

Positive priming bias in pre-service teachers with strong and weak professional identity was calculated using the following formula: Positive priming bias = the number of positive items generated minus the number of negative items generated (Feng et al., 2008; Wei, 2008). The results are shown in **Figure 2**.

The results of the second ANOVA indicated that the main effects of priming stimulus, *F*(2,80) = 418.44, *p* < 0.001, *partial η^2^* = 0.91, and professional identity, *F*(1,40) = 21.71, *p* < 0.001, *partial η^2^* = 0.35) were significant; however, the interaction between these two variables was not significant, *F*(2,80) = 1.54, *p* = 0.221.

The main objective of the study was to compare the effects of priming involving different types of profession-related events between pre-service teachers with strong and weak professional identity. In accordance with this objective and the results of the priming bias analysis (**Figure 2**), we analyzed the differences in priming bias toward positive, neutral, and negative stimuli between pre-service teachers with strong and weak professional identity. The results indicated that pre-service teachers with strong professional identity showed significantly greater positive priming bias toward positive stimuli compared to that observed in those with weak professional identity, *t*(40) = 4.14, *p* < 0.001, *Cohen’s d* = 1.31. Pre-service teachers with strong professional identity showed significantly greater positive priming bias toward neutral stimuli, relative to that observed in those with weak professional identity, *t*(40) = 3.21, *p* < 0.01, *Cohen’s d* = 1.02. In addition, pre-service teachers with weak professional identity showed significantly greater negative priming bias toward negative stimuli relative to that observed in those with strong professional identity, *t*(40) = 3.06, *p* < 0.01, *Cohen’s d* = 0.97. Therefore, pre-service teachers with strong professional identity showed greater positive priming bias toward positive and neutral stimuli, while pre-service teachers with weak professional identity showed greater negative priming bias toward negative stimuli.

## Discussion

### Differences in Priming Effects According to Participants’ Professional Identity

Research examining professional identity in pre-service teachers is currently progressing rapidly both in China and abroad. Nevertheless, the methodology used in this research has been limited mainly to theoretical discourse and surveys. Very few studies have used experimental methods to explore the internal psychological mechanisms underlying the development of professional identity in pre-service teachers, and even fewer have examined social information processing in pre-service teachers with different levels of professional identity. The current study performed a comprehensive examination of differences in priming effects between pre-service teachers with strong and weak professional identity via the priming paradigm used in Tversky’s social cognition experiment (Tversky and Marsh, 2000; Feng et al., 2008; Wei, 2008). The results showed that, in priming conditions involving different types of stimuli, pre-service teachers with strong professional identity generated a higher number of positive items, relative to that observed in those with weak professional identity, indicating positive priming bias. In addition, in the priming condition involving negative stimuli, pre-service teachers with weak professional identity generated a higher number of negative items, relative to that observed in those with strong professional identity, indicating negative priming bias. There are two possible reasons for this difference. First, the impact of stimulus type on individual behavior is manifested via individuals’ cognition (Brown et al., 2010; Müller et al., 2014; Zhang and Mi, 2015), and once the internal identity mechanism is activated in this manner, individuals perceive their surroundings using their existing knowledge structure; that is, they avoid material and environments that are inconsistent with their identities (Chen et al., 2007) and approach activities and events that are consistent with their identities (Reed, 2004). Therefore, the priming stimuli could have evoked the participants’ internal professional identities, causing them to pay greater attention to information that was consistent with their individual identities, avoid information that was inconsistent with their identities, and exhibit attentional bias. This suggests that the strength of pre-service teachers’ professional identity influenced their attentional bias and the priming effects involving profession-related events.

Second, as pre-service teachers with strong professional identity exhibit stronger personal identity and higher expectations, they could have established self-schemas related to their future teaching profession, which would have influenced their subjective understanding and comprehension of profession-related events. Social schema theory suggests that individuals use different schemas to understand their environments and are inclined to process information that is similar to self-referential schemas and structures (Le, 2004; Bai et al., 2013; Simons et al., 2014). In addition, the question as to which information will play a key role depends on the individual’s attentional threshold values (Bradley et al., 1997; Bai et al., 2013). Pre-service teachers with strong professional identity have a stronger sense of identity within the teaching profession and are willing to work toward becoming excellent teachers in the future. Therefore, when they were primed with profession-related events in teaching, their threshold values for activation could have been lower, leading them to adopt positively primed processing to comprehend and interpret the events experienced by teachers. In contrast, pre-service teachers with weak professional identity might have been unwilling to become teachers when faced with challenges or negative profession-related events and adopted negatively primed processing to comprehend and interpret the events experienced by teachers.

### Differences in Priming Bias According to Participants’ Professional Identity

This result also showed that, when primed with positive and neutral stimuli, pre-service teachers with strong professional identity displayed greater positive priming bias relative to that observed in those with weak professional identity. In contrast, when primed with negative stimuli, pre-service teachers with weak professional identity displayed greater negative priming bias relative to that observed in those with strong professional identity. The characteristics demonstrated in these results differ from the features of priming bias toward profession-related events observed in in-service secondary and primary school teachers (Wei, 2008). That is, pre-service teachers exhibited positive priming bias when primed with neutral stimuli and did not display the negative priming bias observed in in-service secondary and primary school teachers. This implies that the formation of professional identity in teachers is a relatively stable but evolving process (Trent, 2011; Izadinia, 2013; Pillen et al., 2013). Pre-service teachers are still in the preliminary stages of the development of professional identity (Sutherland et al., 2010; Izadinia, 2013). Therefore, they exhibit greater idealism regarding the teaching profession relative to that observed in in-service secondary and primary school teachers, and this manifested as positive priming bias toward stimuli involving neutral profession-related events, even in pre-service teachers with weak professional identity, in the current study. Alternatively, the difference observed could indicate that although pre-service teachers currently receiving teacher training, they have not developed professional identities that are entirely consistent with those of in-service teachers, and instead only use the methods of social information processing used by in-service teachers.

The priming bias observed in pre-service teachers with strong professional identity could have been related to phenomena observed in previous research. For example, some studies have shown that during social information processing, individuals selectively attended to, extracted, and reconstructed events, to preserve consistency with their intrinsic traits (Marchetti, 2014; Keane et al., 2015). In addition, another study demonstrated a high level of test-retest reliability for pre-service teachers’ professional identity (i.e., their professional identity was relatively stable; Wang et al., 2011); therefore their professional identity could be considered a relatively stable professional attitude with a high level of value internalization, similar to a personal trait. Relative to those with weak personal identity, pre-service teachers with strong professional identity are more enthusiastic and willing to actively engage in teaching, which could strengthen their internal professional attitudes. This inherent trait could cause pre-service teachers with strong professional identity to allocate processing resources to positive profession-related events, while selectively transforming negative profession-related events, thereby forming a virtuous cycle in their teaching careers. This could also influence pre-service teachers’ job satisfaction when they begin teaching (Zhao and Li, 2013), causing them to display similar features in social information processing to those observed in in-service secondary and primary school teachers (Wei, 2008).

In addition, it could explain the higher levels of job satisfaction and well-being and lower levels of turnover intention observed in teachers with strong professional identity, relative to those observed in teachers with weak professional identity within the same working environment. Pre-service teachers with weak professional identity lack enthusiasm for teaching and exhibit greater negative priming bias toward profession-related events, relative to that observed in those with strong professional identity. This could lead to a vicious cycle in their teaching careers, which could ultimately cause them to resign from the profession or experience psychological crisis after they begin teaching. Therefore, the scientific identification of pre-service teachers with weak professional identity has important practical implications for education.

### Limitations and Future Research

The present study also has some limitations. First, all subjects were pre-service female teachers, so the results maybe not explain overall teacher professional identity or extend to pre-service male teachers. Hence, more representative samples of teachers should be obtained in future research to verify the results. Second, our study found that pre-service teachers with strong professional identity were likely to have established positive self-schemas involving profession-related events. One study showed that emotion was an appropriate lens to understand better the impact of reform and change on teacher professional identity (Reio, 2005). Thus, psychopathological features (such as anxiety or depression) need to be explored in future studies.

### Practical Implications for Education

If education administrators assess individual pre-service teachers directly using the Professional Identity Scale (Wang et al., 2010), there is a substantial risk of socially desirable or false responses, as it involves respondents’ vital interests. Therefore, education administrators should examine differences in the effects of priming involving profession-related events in pre-service teachers, to differentiate indirectly between those with strong and weak professional identity. This would decrease the probability of false responses and improve the validity of assessments implemented by education administrators, thereby identifying individuals with weak professional identity before they become students or future teachers. With respect to those who are already pre-service teachers, targeted counseling with supportive scaffolding, such as that designed to correct cognitive biases observed in pre-service teachers with weak professional identity (Huang and Wang, 2015; Wang et al., 2017), should be provided to improve the professional competence of future teachers.

## Conclusion

The results of the study showed that, relative to those with weak professional identity, pre-service teachers with strong professional identity showed higher levels of positively primed processing; generated a higher number of positive items when primed with positive, neutral, and negative stimuli; and exhibited greater positive priming bias toward positive and neutral stimuli. In contrast, relative to those with strong professional identity, pre-service teachers with weak professional identity showed higher levels of negatively and neutrally primed processing; generated higher numbers of neutral and negative items when primed with positive and negative stimuli, respectively; and exhibited greater negative priming bias toward negative stimuli.

## Ethics Statement

This study was carried out in accordance with the recommendations of Jiangxi Normal University (School of Psychology)’s ethics committee of guidelines. All subjects gave written informed consent in accordance with the Declaration of Helsinki. The protocol was approved by the Jiangxi Normal University (School of Psychology)’s ethics committee.

## Author Contributions

X-qW: design of the study, data analysis, and paper writing and revising. J-cZ: data collection, data analysis, and paper writing and revising. X-yC: data collection and revising. LL and J-yH: data collection and data analysis.

## Conflict of Interest Statement

The authors declare that the research was conducted in the absence of any commercial or financial relationships that could be construed as a potential conflict of interest.
