# Supplementary material for: Active Construction of Profession-Related Events: The Priming Effect among Pre-service Teachers with Different Professional Identity
Source: Front Psychol. 2018 Feb 27;9:233. doi: 10.3389/fpsyg.2018.00233 (PMC5835339; doi:10.3389/fpsyg.2018.00233)
Supplement: Supplementary file 1 [file Presentation_1.PDF]

Appendix: Experimental reading material

The Story of Two Colleagues

You are a teacher of a middle school, and you have two collegues who named Zhang and Li. Li is a Chinese teacher. He graduated from a normal training college. He has been working for many years. He is a dependable and experienced teacher. Zhang is a math teacher. He graduated from a famous normal university. He is a young teacher without too many experiences.

On Monday morning, as usual, you all arrived earlier. You started to clean the office. Last week, Zhang was on duty. Zhang was outgoing, but lacked of personal sanitation. The office was full of garbages which Zhang left behind. There was a disgusting smell all around the office. When you cleaned the office, Zhang did not realize that his bad habit brought extra labor to you. Zhang did not feel sorry for his habit at all, which made Li angry.

After cleaned the office, Li watered his potted plant, and then arranged the books on the desk. He signed, "A busy semester will end soon. At the school, I was busy with preparing lessons, having class, correcting students' works and so on. At home, I was busy with doing housework, taking care of children. However, I was less cared about myself." Then, he shook his head with a bitter smile.

Zhang heard Li's words, speechless for a while,and then signed, "I agree with you. I used to dream that I will get some great achievements in my job. However, the rigid life of teacher was so boring. Where is my swinging life ? "

"This job is not easy any more" . After arranging the books, Li lit up a cigarette,

and breathed out a swirl of cigarette smoke, said thoughtfully, "In the beginning, when I just joined in my work, all of my schoolmates and friends told me that the job which I took was the most glorious one in the world. It was made contact with a group of naive children. I got all of their envies. Ha-ha" Li laughed, and then, he shook his head, continued his speech, " However, now everyone of them was better than me. We had a reunion the other day. When I saw my old schoolmates, each one of them were complacent. They were either rich or powerful. Compared with them, I felt especially embarrassed. For a long time, I couldn't regulate my mood."

After hearing what Li said, Zhang started to complain. He put down the book, stepped to Li and said: ' Yeah. We worked hard everyday, but how many wages can we get? It is important that education is increasingly considered by government, public and students ' parents now. On the one hand, it’s a good thing that we can realize the value of our occupation. But on the other hand, indeed, it brings some negative effects. The more important people value the education , the more strict they require teachers. After it ,coming with various inspections. We would be supervised by the government, the educational departments, as well as the parents. We prepare courses, have classes, manage students all day long, which is already a tough task. At the same time, we still have to do any thing possible to deal with the inspections. How annoying! Although our country made great policies, some people just shout them in words or print them on the papers instead of implementing these policies. It puts education in a difficult position. '

“Chap! Pain and happiness originated from heart! ”Hearing this, Zhang also

complained, Li switched the conversation, said, "what you said was right. Nowadays, there may be problem in some respects of our education. But, generally, our education has obtained the unprecedented development in recent years. Compared with past, the treatment for our teachers has been greatly improved. If we only paid attention to the dark side and blamed it, there might be no effects. Sometimes even became worse. The only thing we could get from that was the encouragement of the ignorance of others and our own .In contrast, if we save our energy to change ourselves, and to participate in teaching, we could have better results. It’s impossible to demand the environment adapt to us. Only our adaption to the environment could gradually change our environment."

Zhang looked up to Li, while he just wanted to say something but stopped. To be honest, he highly admired Li as he worked in an active attitude in this age. Although sometimes he complained about the work, as well as flaunting his seniority to younger teachers, he actually got a bunch of achievements in teaching. He was a generous people. He was achieved the admiration of students .

Zhang, this is your chances! "At this moment, another colleague pushed into and said, “this semester, the school would recommend Zhang to attend young teachers’ public lesson competition around city. It would keep you busy for a while. Your passion could finally have a place to exert. To have a better future, you need to work more hard.” “Thank you, this is indeed a good news to me, I must be prepared well, I will be busy! “Said Zhang were filled with joy” .

In the following days, Zhang devoted himself totally to preparing for the public

class .There was no his hearty laughter in the office and no his familiar figure on the basketball court any more. No classe time, he always spent half of the day sitting in front of the computer to search information, preparing lessons and designing PPT. Moreover, in order to solve several technical problems, he often consulted with some people who were proficient in computer. Occasionally, he looked thoughtfully when others came across him at school.

Where there is a will there is a way. Zhang finally got a big reward. He won the first prize in young teachers' public lesson competition around city . He will represent the city to take part in the open class competition in province . After the competition, his passion for life was ignited again! Working for two years, finally ,he found the passion of his life as well as the core of his work!
